# Peer Review of “Selection of the Optimal L-asparaginase II Against Acute Lymphoblastic Leukemia: An In Silico Approach“

**DOI:** 10.2196/33215

**Published:** 2021-09-08

**Authors:** Navneetha Hardikar

**Affiliations:** 1 Department of Biology Queen's University Kingston, ON Canada

*This is a peer-review report submitted for the paper “Selection of the Optimal L-asparaginase II Against Acute Lymphoblastic Leukemia: An In Silico Approach”.*

## Round 1 Review

### General Comments

This paper [1] aimed to investigate whether asnB from *E Coli* and *Erwinia* is the best asparaginase for the therapeutic treatment of acute lymphoblastic leukemia by using asnB sequence of *E Coli* to search for homologous proteins in other bacteria and archaea phyla. The authors mention that asnB with the lowest Michaelis Menten constant (Km) and the lowest immunogenicity is to be considered the most suitable enzyme. A phylogenetic tree was created, after which homology modeling was conducted, followed by docking to identify the binding energies to determine the relationship between binding energy and Km. The technical aspects of the paper are adequately conveyed, and the in silico method is appropriate to answer the question. However, I have a few comments that need to be clarified.

### Specific Comments

#### Major Comments

1. Perhaps, explain what blastp does to provide more insight.
2. It would be beneficial to include a figure that displays the sequence alignment of the query sequence along with the similarity percentage.
3. The discussion is well-written, although could benefit from including other relevant studies/prior works to support your results.
4. Conclusion is relatively weak. Please consider revising it and sufficiently summarizing the Methods and Results.

#### Minor Comments

1. Section 2.2: Please define DOPE and SOAP before abbreviating.
2. Discussion: It is mentioned that only 6 of 10 species could have a Km value assigned to a certain sequence, please mention these 6 species.
3. Discussion: The sentence “Thus it can be predicted that an enzyme with better kinetics that currently commercially available asparaginase can be cloned from Streptomyces species” is a bit ambiguous. Please rewrite this sentence.

## Round 2 Review

### General Comments

The authors have addressed all reviewer comments and improved the manuscript. I have no further comments.
